# Patient Safety and the COVID-19 Pandemic in Germany: A Repeated Population-Based Cross-Sectional Survey

**DOI:** 10.3390/ijerph20010112

**Published:** 2022-12-22

**Authors:** Olga Amberger, Angelina Müller, Dorothea Lemke, Hardy Müller, David Schwappach, Peter Wendt, Michel Wensing, Maria-Sophie Brueckle, Beate S. Müller

**Affiliations:** 1 Institute of General Practice, Goethe University Frankfurt, 60590 Frankfurt am Main, Germany; 2 Techniker Krankenkasse, TK, Unternehmenszentrale, 22305 Hamburg, Germany; 3 Institute of Social and Preventive Medicine (ISPM), University Bern, 3012 Bern, Switzerland; 4 Department of General Practice and Health Services Research, University Hospital Heidelberg, 69120 Heidelberg, Germany; 5 Institute of General Practice, University of Cologne, 50937 Köln, Germany

**Keywords:** patient safety, coronavirus pandemic, PROM

## Abstract

The coronavirus (COVID-19) has presented Germany with major challenges and has led to concerns about patient safety. We conducted an observational, population-based, nationwide, repeated cross-sectional survey on patient safety in Germany in 2019, 2020, and 2021. Each of the three samples consisted of 1000 randomly recruited adults. Self-reported data via computer-assisted telephone interviews were taken from TK Monitor of Patient Safety. Perceptions, experience, and knowledge relating to patient safety were assessed. The majority of respondents considered medical treatment to involve risks to patient safety. This proportion decreased during the pandemic. The majority also had a high degree of self-efficacy regarding the prevention of medical errors, whereby the percentage that felt well informed with regard to patient safety rose throughout the pandemic. The proportion of persons that suspected they had in the past experienced an error in their treatment remained steady at one third as well as the reported errors. In 2020, 65% of respondents thought health communication with service providers (e.g., extent and comprehensibility of information) remained unchanged during the pandemic, while 35% reported that medical appointments had been cancelled or postponed. This study is the first to assess patient safety from a general population perspective during the coronavirus pandemic in Germany. COVID-19 had a positive impact on perceived patient safety but no impact on suspected and reported errors. Self-efficacy with regard to medical error prevention steadily increased in the general population, and people considered themselves well informed.

## 1. Introduction

Since December 2019, the coronavirus disease 2019 (COVID-19) has spread throughout the world, posing major challenges to health systems [1]. The degree to which COVID-19 has required health systems to rapidly redesign the delivery of care has emphasized the high risk of patient safety. Patient safety is an essential feature of care quality and refers to the absence of adverse events resulting from treatment [2]. Patient safety incidents are estimated to rank fourteenth among the causes of morbidity and mortality [3]. Although patient safety remains a priority issue in health policy strategies in Germany [4,5], it continues to be one of the biggest problems in terms of economic burden [6].

The COVID-19 pandemic revealed a range of safety gaps including provision of health services, communication, and management of health information [1]. The magnitude of avoidable harm from the COVID-19 pandemic has not yet been fully assessed, as current systems for measurement of patient safety are not well developed. Data on patient-reported experiences relating to patient safety during the pandemic are herein limited [7,8].

The perceptions, experience, and knowledge of patients complements the views of medical staff, healthcare payers, and other stakeholders in the healthcare system and can make a significant contribution towards improving patient safety [9,10]. Patients are an important source of information, and for some symptoms (e.g., pain intensity) they represent the only source of data [11] because they can proactively identify potential risks to safety. Moreover, only patients are able to report on the effects of treatment across sectors and over long periods of time. The information they provide on incidents may therefore be more accurate than that of physicians [12], who are often reluctant to report patient safety problems [13]. Evaluating patient surveys is now an established method of measuring quality in German inpatient settings [14]. The Techniker Krankenkasse (TK) Monitor of Patient Safety annually collects population-related data on perceptions, experience, and knowledge pertaining to patient safety from 1000 randomly selected adults [15]. The survey spectrum covers the assessment of risks, error reduction, risk mitigation, avoidance, and management, and its aim is to identify and explore nationwide patterns and trends in patient safety from a population perspective. Every year, the survey is updated to include questions about recent issues. The first survey took place in 2019. Follow-up surveys were undertaken in the following two years. In 2020, questions were added on healthcare and health communication during the coronavirus pandemic, and in 2021, additional items relating to symptoms of so-called ‘long COVID’ were included. In Germany, the first wave of the Corona pandemic lasted from March to early May 2020 and was initially characterised by a lack of protective equipment and the need for improvements in organisation, hygiene, and protection measures in medical and nursing facilities. The subsequent “interim phase” was characterised by the slow and cautious opening of schools and kindergartens that were previously closed during the lockdown and by low overall levels of infection in the general population. From the end of September 2020 until May 2021, the number of COVID-19 infections increased again throughout Germany. This “second wave” resulted in a large number of hospitalisations and deaths. Selected results from the surveys have been published elsewhere for both a scientific audience and the general public [16].

The aim of the present study was to describe trends in public perceptions, experience, and knowledge concerning patient safety in healthcare. Our study particularly aimed to compare perceptions regarding patient safety before and during the coronavirus pandemic.

## 2. Materials and Methods

### 2.1. Study Design

This cross-sectional study obtained data from repeated, national, population-based surveys (TK Monitor of Patient Safety). The TK Monitor of Patient Safety is a monitoring system that was established in 2019 to help understand trends in patient safety from the German public’s point of view. The survey is undertaken yearly. In the present study, we included data from surveys conducted in 2019, 2020, and 2021. The first survey took place from October to November, 2019 and thus before the COVID-19 pandemic. The follow-up surveys were conducted in August, 2020, after the first wave of the pandemic, and in June, 2021, after the second. Reports on this observational study were prepared in accordance with the Strengthening the Reporting of Observational Studies in Epidemiology (STROBE) checklist for reporting observational studies.

### 2.2. Participants

The TK Monitor of Patient Safety uses self-reported data that are collected yearly from 1000 randomly selected adults living in Germany. The survey method has been described elsewhere and will be discussed briefly here [16]. The representativeness of the sample was ensured by drawings of ADM (Arbeitskreis Deutscher Marktforscher, Association of German Market and Social Research Institutes) samples and by comparison with the data of German Federal Statistical Office [17]. The sample was representative for the German community regarding age, gender, educational level, and region. A multi-level stratified random sampling procedure was employed (dual frame design: 70% landline, 30% mobile phone numbers). In 2021, 69.38 million adults were living in Germany, 84.3% of all households had fixed phone number and 85.6% mobile number [18]. In the sample with landline numbers, respondents were selected using the last birthday method. This method is based on the informant accurately acknowledging which person in the household had a birthday last and assumes that the informant is aware of the birth dates of the other adult members of the household. Inclusion criteria were German as a native language and 18 or more years of age. The TK Monitor of Patient Safety uses computer-assisted telephone interviewing (CATI) to assess knowledge, perceptions, and experiences relating to patient safety, and especially to subjectively experienced errors in health care [16]. Data were collected by the Society for Social Research and Statistical Analysis Ltd. (forsa) [19] according to the German law of data protection. Participation in the study was voluntary and no expense allowance was paid. Participants were informed about the anonymity of the survey, the aims of the study, and data protection. They also gave their informed consent. The contact data and quotas including response rate were deleted immediately after the interviews in accordance with data protection regulations. The forsa institute has accepted the code of ethics of the industry association [20].

### 2.3. Questionnaire and Implementation

Due to the absence of a validated CATI survey instrument, the TK Monitor of Patient Safety questionnaire was developed on the basis of literature reviews, preliminary work, and international questionnaires [21,22,23,24,25,26,27,28,29]. The instrument uses closed questions to assess knowledge, perceptions, and experiences relating to patient safety and especially to subjectively experienced errors in healthcare. The questionnaire consists of three sections (see online Supplementary File 1): (A) questions on perceptions, experiences, and subjective information relating to patient safety in medical care. To ensure that they had a common understanding of the term, respondents were first informed that patient safety is understood to mean the avoidance of unintended or unexpected harm to patients during the provision of health care. In section (B), the questions covered perceptions and knowledge regarding COVID, whereby multiple answers were possible. Section (C) collected sociodemographic and socioeconomic data. Section A and C remained almost unchanged every year, while section B changed from survey to survey. In 2020, the questions in section B related to healthcare and health communication during the COVID-19 pandemic, and in 2021 to long COVID. The content of the questionnaire was validated by a panel of experts from different disciplines (social sciences, medicine, health sciences, and psychology) for comprehensibility and consistency. Misleading questions were modified.

### 2.4. Data Analysis

The data were analysed using descriptive statistics. Data were weighted for German age, gender, educational attainment, and area (metropolitan/rural) population distribution. An iterative proportional fitting algorithm was used for weighting. To compare variables among the three surveys, we used a chi-square test. This test is robust with respect to the distribution of the data and does not require equality of variances among the study groups [30]. All tests were performed two-sided at a significance level of alpha = 0.05. Statistical analyses were performed using the R version 4.1 [31].

## 3. Results

### 3.1. Participants

The main sociodemographic characteristics of the three survey samples are presented in Table 1. The data in all three samples were comparable, with the exception of health status, as more respondents rated their state of health as “very good” or “good” during the coronavirus pandemic.

### 3.2. Patient Safety Perceptions from 2019 to 2021

**Figure 1:**
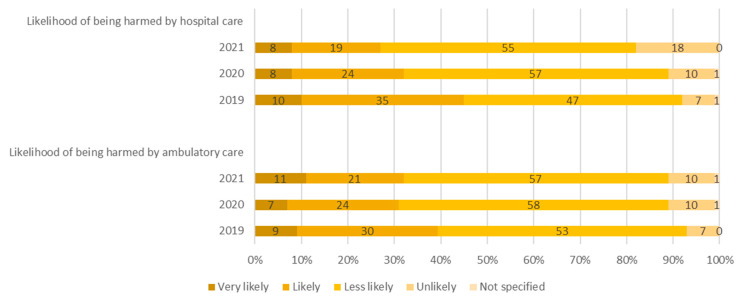
Participants perceptions of the likelihood of being harmed when receiving hospital and ambulatory care, 2019 to 2021 (*n* = 1000 per year).

One quarter to a half of respondents considered it very or somewhat likely that patients would be harmed if they received medical treatment in hospital. The proportion decreased during the coronavirus pandemic (45% in 2019, 32% in 2020, and 27% in 2021: question 2, Figure 1). The same trend was observed in the assessment of risk associated with ambulatory care with slightly lower levels during the coronavirus pandemic (*p* < 0.0001). In 2019, 39%, in 2020, 31%, and in 2021, 32% of respondents considered it very or somewhat likely that patients would be harmed if they received ambulatory care (question 3, Figure 1). With regard to the likelihood of patient safety problems, the majority of respondents considered it very likely that an illness would be diagnosed wrongly (59%, 51%, 53%: multiple choice question 4, Table 2), or that they would contract a nosocomial infection (63%, 56%, 62%), at some stage in their lives. Other sources of error, such as errors during surgery, medication errors, or errors in the use of medical devices, were considered likely by fewer than half the respondents. In 2020, 31% of respondents regarded it as likely they would be infected with coronavirus in hospital or when receiving ambulatory care. This percentage remained steady in 2021 (27%). Two thirds of respondents regarded patient harm as largely preventable if appropriate measures were taken (multiple choice question 5, Table 2). In 2020 and 2021, the majority regarded the prevention of a coronavirus infection in hospital or ambulatory care as likely if appropriate measures were taken. This proportion increased between 2020 and 2021 (55%, 66%).

### 3.3. Experiences with Patient Safety from 2019 to 2021

After suspecting an error, the majority of respondents would contact the attending physician (77% in 2019, 73% in 2020: multiple choice question 6, not applicable in 2021), another physician (81%, 84%), their health insurer (77%, 73%), a patient counselling centre (64%, 58%), or a lawyer (67%, 58%). Three-quarters of respondents indicated moderate to high self-efficacy regarding error prevention, with a higher level during the coronavirus pandemic (69%, 75%, 74%: question 9, Table 3). The majority of respondents rated their knowledge of patient safety as “very good” or “good” (question 8, Table 3) with increased rates during the coronavirus pandemic (55%, 68%, 69%). The proportion that felt less well or not at all well-informed decreased accordingly (45%, 32%, 30%).

One-quarter of respondents reported that a suspected error had occurred once or more during a medical examination or during treatment in the last ten years (question 13, Table 4), with responses remaining stable over the period under review (24%, 24%, 26%) and no significant different response distribution over the years. Of these respondents, only one-third actually reported the error with no statistically significant difference in responses over the years (34%, 30%, 40%: question 14, Table 4), which they did predominantly to the attending physician or hospital (68%), or another physician (43%, 53%: multiple choice question 15). In 2020, respondents were more likely than in the previous year to contact a patient counselling centre or consumer advice centre (2%, 15%) to report an error. To avoid medical treatment errors, the respondents said they would use the following services provided by their health insurer (question 16): specific information provided before medical treatment with the aim of helping avoid treatment errors (2020: 84%), a survey after medical treatment to check whether problems, or a treatment error, had occurred (83%), information on avoiding medical treatment errors (64%), and training courses (41%).

### 3.4. Healthcare during COVID-19 Pandemic (Results from 2020)

Fifteen percent of respondents had been tested for COVID-19 (question 23), of whom 3% were positive (0.4% of all respondents, question 24). Of those that had not been tested, the majority (83%) did not believe they had already had a coronavirus infection (question 25). About 48% of respondents were afraid of being infected (question 27) and 58% were afraid of becoming seriously ill if they were infected with the coronavirus (question 28). Sixty-five percent of respondents said that, during the coronavirus pandemic, health communication with providers had not deteriorated in terms of the amount of information and its comprehensibility (question 18), while one-third of patients (35%) said certain services had been cancelled or postponed during the coronavirus pandemic (question 19), the majority (64%) of them by providers (question 20). In 70% of cases, cancellations by patients were due to fear of a COVID-19 infection, while one-third (38%) justified their cancellation by referring to the burden on the healthcare system (multiple choice question 21). In contrast, fear of receiving poor treatment was not a motive for postponing services (5%). Overall, 9% of respondents deliberately avoided going to a pharmacy (question 22), while the percentages of the following respondents reporting no concerns about using medical facilities were: family practice or specialist 71%; dentist 74%; hospital 48%, therapists (such as a physical therapist) 75%; nursing services 66% (multiple choice question 26).

### 3.5. Knowledge about Long COVID (Results from 2021)

Only three percent of respondents had not heard of any late complications associated with COVID-19. The best known late complications were taste and smell impairment (86%), shortness of breath (85%), persistent fatigue and tiredness (84%), and headaches (74%). Less known complications included chest pain (35%) and recurrent fevers (30%). Most respondents considered primary care physicians (85%) and pulmonary specialists (79%) to be the first point of contact in the care of long COVID-19 patients. Hospitals were less often considered responsible for the care of patients with long COVID-19 (42%).

## 4. Discussion

To best of our knowledge, this is the first study to assess perceptions, experiences, and knowledge relating to patient safety in healthcare among the general population of Germany before and during the COVID-19 pandemic. This study reflects the views of the general public, which may differ from those of medical professionals. The results show trends in patient safety from the perspective of the broader population, and may help in the development of appropriate protective measures.

Our results showed that COVID-19 had a positive impact on the perceived safety of healthcare in Germany but no impact on suspected and reported errors. Although there were lower levels during than before coronavirus pandemic, risks were generally perceived to be high and many participants had had personal experiences of patient safety incidents over the years. Every third respondent considered harm from treatment to be likely both in hospital and in ambulatory settings. One in four respondents suspected a medical error had occurred in the previous ten years. This view remained at similar levels both before and during the COVID-19 pandemic. Although patient safety issues are widespread, this rate is higher than was found in national and international studies before the COVID-19 pandemic [23,32,33]. On the other hand, data on patient-reported experiences relating to patient safety during the course of the pandemic are scarce [7,8]. Country-level analyses based on data from the Commonwealth Fund’s 2010 lnternational Survey of the General Public’s Views of their Health Care System’s Performance in 11 Countries [23] showed the average frequency of patient-reported errors in two years to be 11.2%, but with marked differences between countries, and levels ranging from 5.4% in the United Kingdom to 17.0% in Norway. According to the 2019 OECD report, many indicators of patient safety had improved before COVID-19 [33]. During the COVID-19 period, however, significant deterioration in patient safety was observed in Spanish primary care [7], whereby short recall periods as well as differences in recording methods and healthcare systems should be taken into consideration, which make comparisons difficult.

The majority of respondents in our study believe that the adoption of appropriate measures can prevent patient harm. Indeed, there is evidence that 70% of injuries from medical errors could have been avoided [34]. In agreement with a previous study [35], the majority of respondents to the TK survey indicated moderate to high self-efficacy in error prevention. There is quite broadly shared agreement that some harm in healthcare is inevitable, but this view is not becoming more widespread [36]. COVID-19 has increased waiting times for elective procedures [37], and reduced healthcare utilisation in terms of fewer consultations, admissions, diagnostic investigations, and therapies [38]. Moreover, COVID-19 itself is transmitted in healthcare institutions (nosocomial infection), but this is more likely in nursing homes than in hospitals and ambulatory care [39].

In our study, the frequency of patient-reported errors had remained at a low level over the years and was no different before to during the pandemic. However, general willingness to report errors, particularly to patient and consumer advice centres, increased during the coronavirus pandemic. Low levels of patient-reported error frequency are consistent with data from the Commonwealth Fund’s 2010 lnternational Survey of the General Public’s Views of their Health Care System’s Performance in 11 Countries [23]. Underreporting of patient safety incidents and associated biases, such as identification and reporting biases, reduces the healthcare system’s ability to quantify harm reduction [40]. However, a positive error culture should be a key element in a new systemic safety culture [41]. As the COVID-19 virus has affected healthcare systems, it is especially important to ensure patient safety monitoring systems are robust.

The rate of reported positive test results is in line with the Robert Koch Institute’s figures for the country as a whole [42]. According to our study, one in three respondents reported that services had been cancelled or postponed during the coronavirus pandemic. In international studies, this rate ranged from 20% for postoperative radiation therapy in breast cancer [43] to 88% for paediatric emergency department visits [44]. Moreover, in contrast to other European countries, most of our respondents expressed confidence when using healthcare facilities during the pandemic [45]. Although other studies describe a lack of coordination between different healthcare sectors [46], communication with physicians, therapists, and nurses did not deteriorate as a result of the coronavirus pandemic, according to the TK surveys.

In agreement with the systematic review conducted by Saadatjoo et al. [47], our study showed a relatively high level of knowledge about long COVID-19. However, there are differences in patients’ sources of knowledge and its accuracy [48], which may have encouraged patients to adopt problematic health behaviours.

The following aspects should be taken into account when interpreting the results. Since contact data were deleted immediately after the interviews in accordance with data protection regulations, response rate and reasons for not participating are unknown. The participating population may herein differ from the population that does not participate. However, the sample was representative for the German community and, in our study, the focus was on the subjective views and knowledge of the general public. Subjective knowledge, however, can differ significantly from objective measurements [49]. Furthermore, the public’s views may be influenced by methods used in the survey and associated predetermined response categories. Given the sensitive topic, interviews via CATI may have made the respondents less comfortable and thus more susceptible to the social-desirability bias. Cognitive pretests were not used to check the comprehensibility of questionnaire items, so the possibility that questions were misunderstood cannot be ruled out. Furthermore, the reliability of the measure was not tested which may have affected our results.

A strength of the study is that, for the first-time, data on perceptions, experience, and knowledge pertaining to patient safety were collected in representative public samples before and during the COVID-19 pandemic, so that changes over time could be identified. The methods are applied to other countries [50]. Furthermore, the employed CATI technique prevents measurement errors and item-nonresponse, and the use of reliable filter questions may have helped guide respondents through the questionnaire [51].

Patient-reported approaches that are not cross-checked against comparative data (e.g., record reviews) do not allow objective conclusions to be drawn. However, our findings yield important complementary and potentially actionable safety information which may help healthcare systems to develop targeted measures. Moreover, we followed international recommendations to consider patients’ view as an important source of information, and to actively involve them for recording patient safety problems [13]. Further research is essential to assess the influence of perception, experiences, self-efficacy, and knowledge as predictors of patients’ safety.

## 5. Conclusions

Our results indicate that COVID-19 had a positive impact on perceived patient safety in Germany but no impact on suspected and reported errors over the years. The study revealed a high perception of risk whereby the levels were lower during the pandemic. Over the three-year period, respondents felt increasingly well informed about patient safety and reported high self-efficacy with regard to error prevention. During the pandemic, respondents expressed confidence in the healthcare system. The best known symptoms of long COVID were taste and smell impairment, shortness of breath, persistent fatigue and tiredness, and headaches. Given these insights from an analysis of a large data set, this paper contributes comprehensive evidence on patient safety from a general population perspective. The findings have important implications for patient safety strategies, and may help determine preventive measures. Furthermore, our study indicates that patients are a valuable source to identify patient safety concerns in this and future crises.

## Figures and Tables

**Table 1:** Socio-demographic characteristics from 2019 to 2021.

Demographic	Characteristics	2019 *n* (%)	2020 *n* (%)	2021 *n* (%)
Gender	Male	489 (49)	490 (49)	489 (49)
	Female	511 (51)	510 (51)	511 (51)
Age group	18–39 years	319 (32)	317 (32)	316 (32)
	40–59 years	348 (35)	321 (32)	324 (32)
	≥60 years	333 (33)	362 (36)	360 (36)
Employment status	Employed	524 (52)	551 (55)	537 (54)
	Unemployed	476 (48)	449 (45)	463 (46)
Chronic condition	Yes	366 (37)	311 (31)	287 (29)
	No	634 (63)	689 (69)	713 (71)
Self-rated health status	Very good/good	572 (57)	679 (68)	677 (68)
	Satisfactory	295 (30)	255 (26)	254 (25)
	Less good/bad	133 (13)	66 (7)	69 (7)
Number of prescription medications	No	458 (46)	508 (51)	533 (53)
	One drug	177 (18)	211 (21)	175 (18)
	Two drugs	133 (13)	117 (12)	111 (11)
	Three or more drugs	231 (23)	164 (16)	181 (18)
Education level	Primary school	338 (34)	83 (8)	65 (7)
	Secondary school	280 (28)	258 (26)	255 (23)
	Tertiary/University	355 (36)	637 (64)	663 (66)
Insurance status	Statutory health insurance	875 (88)	786 (79)	807 (81)
	Private health insurance	125 (13)	214 (21)	193 (19)

**Table 2:** Participants’ perceptions of the likelihood of adverse events and their prevention, 2019 to 2021 (*n* = 1000 per year).

		Likelihood of Adverse Events				Likelihood of Prevention of Adverse Events			
		2019	2020	2021	*p* Value	2019	2020	2021	*p* Value
Hospital-acquired infection					<0.0001				<0.0001
	Yes, definitely (%)	16	12	15		21	13	13	
	Yes, probably (%)	47	44	47		43	47	54	
	Probably not (%)	31	35	33		29	33	27	
	Certainly not (%)	6	8	4		7	6	6	
	Not specified (%)	1	1	2		0	0	0	
Wrong diagnosis					0.001				0.8912
	Yes, definitely (%)	14	14	13		17	15	16	
	Yes, probably (%)	45	36	40		42	44	45	
	Probably not (%)	36	42	42		34	34	33	
	Certainly not (%)	5	6	5		6	6	6	
	Not specified (%)	0	1	0		1	1	1	
Error during operation					<0.0001				0.0823
	Yes, definitely (%)	6	7	8		16	14	15	
	Yes, probably (%)	36	24	27		39	45	43	
	Probably not (%)	51	55	55		36	31	33	
	Certainly not (%)	7	13	9		7	8	8	
	Not specified (%)	0	1	1		1	1	1	
Medication error					<0.0001				0.0018
	Yes, definitely (%)	8	8	12		20	19	19	
	Yes, probably (%)	40	30	32		42	37	45	
	Probably not (%)	43	48	45		32	36	29	
	Certainly not (%)	9	13	11		5	8	6	
	Not specified (%)	0	0	0		1	1	1	
Medical device adverse event					<0.0001				0.1298
	Yes, definitely (%)	5	3	3		18	20	18	
	Yes, probably (%)	26	20	21		41	40	47	
	Probably not (%)	58	62	62		31	30	28	
	Certainly not (%)	12	14	12		7	8	6	
	Not specified (%)	0	1	2		2	2	2	

**Table 3:** Participants’ perceptions relating to self-efficacy in error prevention and knowledge about patient safety, 2019 to 2021 (*n* = 1000 per year).

	2019	2020	2021	*p* Value
Self-efficacy in error prevention				<0.0001
Yes, definitely (%)	26	34	34	
Yes, probably (%)	43	41	40	
Probably not (%)	23	20	18	
Not at all (%)	6	5	8	
Not specified (%)	2	0	0	
Patient safety knowledge				<0.0001
Good (%)	9	11	18	
Moderate (%)	46	57	51	
Poor (%)	34	24	23	
None at all (%)	11	8	8	
Not specified (%)	0	0	1	

**Table 4:** Suspected and reported errors, 2019 to 2021.

		2019	2020	2021	*p* Value
Suspected errors					0.2238
	All respondents	1000	1000	1000	
	Yes, once (%)	17	18	18	
	Yes, several times (%)	7	6	9	
	No (%)	75	76	73	
	Not specified (%)	0	0	0	
Reported errors					0.1648
	Respondents reporting an error	242	237	266	
	Yes (%)	34	30	40	
	No (%)	64	67	57	
	Not specified (%)	2	2	3	

## Data Availability

No additional data are available.

## Supplementary Materials

The following supporting information can be downloaded at: https://www.mdpi.com/article/10.3390/ijerph20010112/s1, Supplement 1: Questionnaire, Supplement 2: STROBE checklist.
