# Supplementary material for: Patient Safety and the COVID-19 Pandemic in Germany: A Repeated Population-Based Cross-Sectional Survey
Source: Int J Environ Res Public Health. 2022 Dec 22;20(1):112. doi: 10.3390/ijerph20010112 (PMC9819909; doi:10.3390/ijerph20010112)
Supplement: Supplementary file 1 [file ijerph-20-00112-s001.zip › Supplement 1-Questionaire.pdf]

Questionnaire TK Monitor of Patient Safety 2021

Part 1
Standard introduction

Question S1
What is your year of birth?

Question S2
Interviewer entry: Gender of the interviewee(s).

1 Male
2 Female
3 Diverse

Question A
Health insurance
What is the name of your statutory health insurance provider? Or do you have private health insurance?
INT.: DO NOT READ ALOUD. ONLY ONE NOMINATION.

1 Private health insurance (PKV, no matter what health insurer)
2 AOK (Allgemeine Ortskrankenkasse)
3 Barmer (also: Barmer GEK, BEK)
4 BKK – health insurance provider for employees at certain companies (regardless which)
5 DAK – (Deutsche Angestellten-Krankenkasse, also DAK Health)
6 IKK – health insurance provider for employees at certain companies (regardless which)
7 KKH (Kaufmännische Krankenkasse)
8 Knappschaft
9 TK - Techniker Krankenkasse
10 Another statutory health insurance provider (i.e. NOT privately insured)
11 Do not know/ not specified ➔ END, no interview

Filter: only for persons with statutory health insurance (see question A items 2-10)
Question B
Health insurance status
And what is your health insurance status: Are you compulsorily insured, voluntarily insured or co-insured as a family member with your health insurance fund?
INT.: ONLY ONE NOMINATION.

1 Compulsory health insurance
2 Voluntary insurance (has statutory health insurance despite fulfilling financial requirements to be eligible for private health insurance)
3 Insured as a family member
4 Do not know/ not specified

To all

Question 1 (not applicable in 2020 and 2021)

When you think of the term 'patient safety', which of the following aspects do you think are being referred to?

1 ... Protection against fire in hospital or at the doctor's surgery
2 ... Protection against theft in hospital or at the doctor's surgery
3 ... Protection against misuse of patient data, e.g. unauthorised disclosure of diagnostic data to third parties
4 ... Protection against receiving the wrong medical treatment
5 ... Protection from complications during medical treatment
6 SPONTANEOUS: Other, namely: ....
7 do not know/ not specified

Question 2

'Patient safety' is understood to mean the avoidance of unintended or unexpected harm to patients during the provision of health care.

How likely do you think it is that patients in Germany will come to harm as a result of medical treatment in hospital: Do you think it is 'very likely' - 'somewhat likely' - 'as liely as not' - or 'unlikely'?

1 very likely
2 somewhat likely
3 as likely as not
4 unlikely
5 do not know/ not specified

Question 3

And how likely do you think it is that patients will be harmed by medical care provided outside hospital in Germany, e.g. when being treated as an outpatient at a doctor's surgery, or as a result of taking the wrong medication? Do you think it is 'very likely' - 'somewhat likely' - 'as likely as not' - or 'unlikely'?

1 very likely
2 somewhat likely
3 as likely as not
4 unlikely
5 do not know/ not specified

Question 4

I will now list possible harmful incidents in medical care, also called 'adverse events'.
Please tell me in each case whether you expect this to happen to you one day: 'yes, definitely' - 'yes, probably' - 'probably not' - or 'definitely not'?
How likely is it that the following will happen to you: ...?
INT.:REPEAT SCALE IF NECESSARY.
PRO.: RANDOMISE. BUT a1) ALWAYS DIRECTLY BEFORE a)

a1) ... An infection with the Corona virus at the doctor's surgery or in hospital
a) ... A dangerous bacterial infection in a hospital
b) ... A wrong diagnosis
c) ... An error during an operation, e.g. a surgical error
d) ... A medication error due to the wrong prescription, dosage or method of application
e) ... An adverse event caused in the use of a medical device.

Scale:

1 yes, definitely (incl. has already happened to me)
2 yes, probably
3 probably not
4 definitely not
5 do not know/ not specified

Question 5
I will now read the list of 'adverse events' to you again. Please tell me now in each case whether you think that by taking the appropriate measures, such events could be largely avoided in the future: 'yes, definitely'
- 'yes, probably' - 'probably not' - or 'definitely not'?
Could the following be largely avoided in the future...? INT.: REPEAT SCALE IF NECESSARY.
PRO.: RANDOMISE. BUT a1) ALWAYS DIRECTLY BEFORE a)

a1) ... An infection with the Corona virus at the doctor's or in hospital
a) ... A dangerous bacterial infection in a hospital
b) ... A wrong diagnosis
c) ... An error during an operation, e.g. a surgical error
d) ... An medication error due to the wrong prescription, dosage or method of application
e) ... An adverse event caused in the use of a medical device.

Scale:
1 yes, definitely
2 yes, probably
3 probably not
4 definitely not
5 do not know/ not specified

Question 6 (not applicable in 2021)
Who would you contact if you thought a mistake had been made in your healthcare?

1 ... The attending physician or hospital
2 ... Another doctor (e.g. general practitioner if he or she was not involved in your treatment).
3 ... A medical association
4 ... Your statutory or private health insurance provider, as applicable
5 ... A patient counselling centre or patient organisation
6 ... A consumer advice centre
7 ... A lawyer
8 SPONTANEOUS: Other, namely: ....
9 do not know/ not specified

Question 7 (not applicable in 2020 and 2021)
Health insurance companies have the possibility - based on the data available to them for billing - to recognise whether you, as a patient, have been prescribed medication that could lead to 'adverse events'.

Would you like to be notified by your health insurance company in such a case - or is this not necessary because, for example, the package insert provides sufficient information on side effects?

1 I would like to be notified by my health insurance provider
2 No notification is necessary
3 Do not know/ not specified

Question 8
We have now talked a bit about 'patient safety'. How well informed do you feel you are about patient safety in general: 'very well' - 'well' - 'acceptably well' - or 'not at all'?

1 very well
2 well
3 acceptably well
4 not at all
5 do not know/ not specified

Question 9
All in all, do you think that as a patient, you can contribute towards improving the care you receive at the doctor's surgery or in hospital: 'definitely' - 'probably' - 'perhaps' - 'not at all'?

1 definitely
2 probably
3 perhaps
4 not at all
5 do not know/ not specified

Question 10 (not applicable in 2020 and 2021)
In the last 12 months, has a pharmacist or doctor checked and discussed with you the effects and side effects of all the different prescription medicines you are taking?

1 yes, all of them
2 yes, but not all of them (only the new ones/only some of them ...)
3 no
4 SPONTANEOUS: I do not take a prescription medication (regularly)
5 do not know/ not specified

Question 11 (not applicable in 2020 and 2021)
Do you have an up-to-date list of all the prescription medicines you are currently taking?

1 yes, I have an up-to-date list of all my prescription medicines.
2 yes, but it is not quite up-to-date/not all of them are on it
3 no
4 SPONTANEOUS: I do not take a prescription medication (regularly)
5 do not know/ not specified

Question 12 (not applicable in 2020 and 2021)
In the last 10 years, have you ever received the wrong medication or the wrong dose - whether from a doctor, in hospital or at a pharmacy?

1 yes
2 no
3 I am unsure/ do not know/ not specified

Question 13
Not including dental treatment: Thinking back over the last 10 years, have you ever suspected an error had been made during a medical examination or when receiving treatment from a doctor, nurse or another health professional?
INT.: If 'YES' ASK: Once or several times?

1 yes, once
2 yes, several times

3 no
4 do not know/ not specified

Filter: Only ask if a medical error has occurred according to question 13, item 1 or 2.

Question 14

PRO: FALLS LT. F13, POS. 2 'MULTIPLE': And if you think specifically of the last time you suspect a medical error was made:
PRO: TO ALL LT. FILTER
Did you or a member of your family report the mistake?

1 yes
2 no
3 I am unsure/ do not know/ not specified

Filter: Only ask if response to question 14 was positive.

Question 15

And who did you report the error to? Was that ...
INT.: QUERY INDIVIDUALLY. MULTIPLE NOMINATIONS POSSIBLE.
PRO: FIXED ORDER.

1 ... The attending physician or the hospital
2 ... Another doctor (e.g. general practitioner, if he/she was not involved in treatment).
3 ... A medical association
4 ... Your statutory or private health insurance provider
5 ... A patient counselling centre or patient organisation
6 ... A consumer advice centre
7 ... A lawyer
8 SPONTANEOUS: Other, namely: ....
9 Do not know/ not specified

Question 16 (not applicable in 2019 and 2021)

[if GKV:] health insurers [if PKV:] health insurers can support their insurees in various ways to prevent medical treatment errors. If your [if GKV:] health insurance [if PKV:] health insurance offered this - would you want to use the following offers: 'yes, definitely', 'yes, probably', 'probably not' or 'definitely not'?
Would you want to use the following...:
INT.: REPEAT SCALE IF NECESSARY.
PRO.: FIXED ORDER.

a)... before medical treatment, specific information from your [if GKV:] health insurance [if PKV:] health insurance about what you yourself can do as a patient to prevent treatment errors.
b)... following medical treatment, a questioning by your [if GKV:] health insurance [if PKV:] health insurance as to whether there were problems or even a treatment error.
c)... Training offers from your [if GKV:] health insurance [if PKV:] health insurance on how to behave safely in hospital or at the doctor.
d)... information from your [if GKV:] health insurance [if PKV:] health insurance on the topic of 'Prevention of medical treatment errors', i.e. e.g. in the member magazine, on the homepage or in the app.

Scale:
1 yes, definitely
2 yes, probably
3 probably not
4 definitely not
5 do not know/ not specified

Question 17 (not applicable in 2019 and 2021)
Please think about your contacts with doctors, therapists or nurses now.
During the corona pandemic.
Compared to the time before, was your communication with these medical personnel during the corona pandemic overall 'better' than before corona - 'unchanged' - or 'worse'?

1 better than before the corona pandemic
2 unchanged
3 worse than before the corona pandemic
4 SPONTANEOUS: had no contact/ do not know/ not specified

<u>Filter: Only ask if in question 17 not pos. 4</u>
Question 18 (not applicable in 2019 and 2021)
And in detail? Please tell me also for each of the following aspects whether this was all in all 'better' during the corona pandemic than before corona - 'unchanged' - or 'worse'.
How was your communication with doctors, therapists and nurses during the corona pandemic compared to the time before with regard to ...?
INT.: REPEAT SCALE IF NECESSARY.
INT:. ON REQUEST: IN CASE OF SEVERAL TREATMENTS, REFER TO THE MOST SIGNIFICANT ONE.
INT.: REPLY IF NEEDED: If you have not had experience with an aspect during Corona, please tell me that too.
PRO.: RANDOMISE.

a)... the amount of information you were given
b)... the comprehensibility of the information given to you
c)... the timely arrangement of further treatment steps
d)... the consideration of your personal situation
e) the consideration of your concerns and fears
f)... asking if you have understood everything

<u>Scale:</u>
1 better than before the corona pandemic
2 unchanged
3 worse than before the corona pandemic
4 do not know/ no experience with it during corona pandemic

<u>To all</u>
Question 19 (not applicable in 2019 and 2021)
For each of the following medical services, please tell me now - simply by saying 'yes' or 'no' - whether it was provided to you personally during the corona pandemic, but had been subsequently cancelled or postponed during the corona pandemic - whether by yourself or by a doctor, therapist or hospital.
INT.: QUERY INDIVIDUALLY. MULTIPLE NOMINATIONS POSSIBLE.
PRO.: FIXED ORDER.

1... Treatment cancelled or postponed due to corona pandemic with the GP.
2... Treatment cancelled or postponed due to corona pandemic with the specialists
3... Treatment cancelled or postponed due to corona pandemic with the dentist
4... Treatment cancelled or postponed with the therapist due to corona pandemic, e.g. physiotherapy
5 Treatment cancelled or postponed at the hospital because of corona pandemic
6 Care cancelled or postponed due to corona by a nursing service
7 DO NOT PRE-read: none of this was planned/ none was cancelled/ postponed.

8 Do not know/ not specified

Filter: Ask only for services that were cancelled/postponed according to question 19.

Question 20 (not applicable in 2019 and 2021)

And WHERE were these appointments cancelled or postponed in each case? By 'yourself' - or by the respective doctor, therapist or hospital?
Who was decisive for the cancellation or postponement: ...?
INT.: REPEAT IF NEEDED: yourself - or the doctor, therapist or hospital?...
PRO: FIXED ORDER.

a)... of your planned treatment with the GP
b)... of your planned treatment with the specialist
c)... of your planned treatment with the dentist
d)... your planned treatment with the therapist, e.g. physiotherapy
e)... your planned treatment at hospital
f)... of your planned care by a nursing service.

Scale:
1 Self-cancelled
2 Cancelled by doctor, therapist, hospital
3 Do not know/ not specified

Filter: Directly afterwards, ask separately for each cancelled/ postponed service according to question 20 (item 1).

Question 21 (not applicable in 2019 and 2021)

And which of the following reasons were decisive for you yourself due to the corona pandemic?
Just tell me each time with 'yes' or 'no'.
INT.: QUERY INDIVIDUALLY. MULTIPLE NOMINATIONS POSSIBLE.
PRO.: FIXED ORDER.

a)... have you foregone your planned treatment at the hospital?
b)... have you foregone your planned treatment with the specialist?
c)... have you foregone your planned treatment with the dentist?
d)... have foregone your planned treatment with the therapist, e.g. physiotherapy?
e)... have you dispensed with your planned treatment at the hospital?
f)... you have waived your planned care by a nursing service?

1... because you were afraid of contracting the corona virus
2... because you wanted to relieve the health system
3... because you were afraid of worse treatment
4... because you had a cold yourself or did not want to infect anyone else.
5 SPONTANOUS: other reason, namely: __________
6 do not know/ not specified

To all

Question 22 (not applicable in 2019 and 2021)

Now think about a visit to a pharmacy: have you consciously decided not to go to the pharmacy during corona pandemic?

1 yes
2 no
3 do not know/ not specified

Filter: Only if F22 states that a visit to the pharmacy was deliberately avoided.
Question 22a (not applicable in 2019 and 2021)
And which of the following reasons were decisive for you personally to refrain from visiting the pharmacy during corona pandemic?
INT.: QUERY INDIVIDUALLY. MULTIPLE NOMINATIONS POSSIBLE.
PRO.: FIXED ORDER.

1... because you were afraid of beeing infected with the coronavirus.
2... because you wanted to relieve the health system
3... because you were afraid of worse advice
4... because you had a cold yourself or did not want to infect anyone else.
5 SPONTANOUS: other reason, namely: _________
6 do not know/ not specified

To all
Question 23 (not applicable in 2019 and 2021)
Have you ever been tested for the coronavirus yourself?

1 yes
2 no
3 do not know/ not specified

Filter: Only if according to F23 'yes, tested for coronavirus'.
Question 24 (not applicable in 2019 and 2021)
And did your test been positive for the coronavirus, thus were you infected?

1 yes
2 no
3 do not know/ not specified

Filter: Only if not tested positive for corona according to F24, item 2 or not tested at all according to F23, item 2.
Question 25 (not applicable in 2019 and 2021)
Do you think you have already had a corona infection, even though you have not yet tested positive for the coronavirus?

1 yes
2 no
3 do not know/ not specified

To all
Question 26 (not applicable in 2019 and 2021)
In the current corona situation, how much concern would you have about using the following medical facilities, which I will name in a moment. Would you have 'major concerns' - 'minor concerns' or 'no concerns at all'?
How big would your concerns be ...
INT.: REPEAT SCALE IF NECESSARY:
PRO.: FIXED ORDER.

a)... to go to a GP or specialist practice?
b)... to go to the dentist?
c)... to go to hospital?
d)... to use a therapist, such as a physiotherapist?
e)... to use a nursing service?

Scale:
1 major concerns
2 slight concerns
3 no reservations
4 do not know/ not specified

Question 27 (not applicable in 2019 and 2021)
Are you currently afraid of being infected with the coronavirus: very afraid - quite afraid - or not afraid?

1 yes, very afraid
2 yes, quite afraid
3 no, not afraid
4 do not know/ not specified

Question 28 (not applicable in 2019 and 2021)
And are you currently afraid of becoming seriously ill if you were infected with the coronavirus: very afraid - quite afraid - or not afraid?

1 yes, very afraid
2 yes, quite afraid
3 no, no afraid
4 do not know/ not specified

Special module: Long-Covid

<u>To all</u>

[Rationale of the Long-Covid-Questionnaire: to assess the level of awareness of the problem in order to estimate the need to raise awareness in the general population -> also a task for health insurance funds].

Question LC1
When they are infected with Corona, some people may become ill for a longer period of time. What late symptoms have you heard of?
INT.: QUERY INDIVIDUALLY. MULTIPLE NOMINATIONS POSSIBLE.
PRO.: RANDOMISE

1 Persistent fatigue and exhaustion
2 Headaches
3 Problems concentrating
4 Shortness of breath
5 Taste and smell impairment
6 Persistent cough
7 Recurrent fevers
8 Muscle and joint pain
9 Chest pain
10 Depression
11 None of them
12 do not know/ not specified

Question LC2
From your point of view, who is responsible for the care of people with long-term health problems following a Corona infection?
INT.: QUERY INDIVIDUALLY. MULTIPLE NOMINATIONS POSSIBLE.

PRO: FIXED ORDER.

1 ... The general practitioner or family doctor
2 ... Another doctor (e.g. lung specialist)
3 ... The specialist outpatient clinic of a hospital
4 ... Your statutory or private health insurance provider
5 ... A patient counselling centre or patient organisation
6 SPONTANEOUS: Other, namely: ....
7 Do not know/ not specified

Finally, we have a few statistical questions that we need to evaluate the results.

Question S1/age
Brought forward for structural control.

Question S2/gender
Brought forward for structural control.

<u>To all</u>
Question S3a
If you now think about the overall state of your health: How would you describe your health in general, as 'very good' - 'good' - 'satisfactory' - 'poor' - or as 'very poor'?

1 very good
2 good
3 satisfactory
4 poor
5 very poor
6 do not know/ not specified

Question S3b
Are you receiving regular medical treatment for a serious chronic illness?

1 yes
2 no
3 do not know/ not specified

Question S3c
Do you regularly take a prescription medication?
INT.: IF 'yes' QUESTION: And how many different medicines do you take?

1 1 prescription drug regularly
2 2 prescription drugs regularly
3 3 or more prescription drugs regularly
4 do not know/ not specified

Question S4
Are you currently employed? Gainful employment is defined as any paid activity or activity associated with an income, regardless of the amount of time it takes. Are you...
INT.: READ OUT CATEGORIES INDIVIDUALLY AND COMPLETELY, ALWAYS ASK THROUGH ALL CATEGORIES IF UNCLEAR.

1 Fully employed
2 Employed part-time
3 Semi-retired
4 In marginal employment (so-called mini-job)
5 Working in connection with receiving a jobseekers’ allowance (so-called "one-euro job"

as a condition of receiving ALG II)
6 Employed occasionally or irregularly
7 In vocational training/apprenticeship
8 Retraining
9 In military service/civilian service
10 Economically inactive

Filter: Only if the answer to item 2, 4, 6, 8, or 10 of question S4 was positive

Question S5

Please tell me which of the following groups you belong to.
INT.: READ OUT CATEGORIES ONE BY ONE.

1 Pupil at school
2 Student
3 Pensioner, retiree, early retirement
4 Unemployed
5 Housewife, househusband
6 On maternity leave, parental leave, or other leave of absence
7 Not gainfully employed for other reasons
8 None of the above
9 Do not know/ not specified

To all

Question S6

Educational attainment: What is your highest educational certificate, diploma or degree?

1 No secondary/vocational school leaving certificate
2 Secondary/elementary school leaving certificate
3 Secondary school leaving certificate (Realschulabschluss, Fachschulreife)
4 Completion of polytechnic secondary school
5 Advanced technical college entrance qualification, completion of a specialised upper secondary school
6 ‘A’ levels, or subject-linked higher education entrance qualification (Abitur)
7 University
8 Another school-leaving qualification

Filter: Only if "ACQUIRED".

Question S7

What is your main occupation?

1 Self-employed farmer or cooperative farmer
2 Freelancer
3 Self-employed (trade, craft, industry, service)
4 Civil servant, judge, professional soldier
5 Employee(s)
6 Worker(s)
7 In training
8 Caring for family member(s)
9 Other

To all

Question S8a

How many people live permanently in your household, including yourself? Please also consider children living in your household.

/___/ persons

Filter: Only to multi-person households
Question S8b/Children in the household
How many children under 18 live in your household?

/___/ Children

To all
Question S9
What is your marital status? Are you ...
INT.: PRE READING.

1 ... Married and living with your spouse
2 ... Married and living separately from your spouse
3 ... Single
4 ... Divorced
5 ... Widowed
6 no answer

Filter: Only if response to S9 is not 1 'lives with spouse'.
Question S10
Do you live with a partner in your household?

1 yes
2 no
3 no answer

To all
Question S11
What is the total monthly net income of your household? This is the sum of wages, salaries, income from self-employment, pensions or retirement pensions, in each case after the deduction of taxes and social security contributions. Please also add income from public benefits, income from renting, leasing, housing benefit, child benefit and other income.
INT.: DO NOT READ OUT CATEGORIES

1 under 500 euros
2 500 euros to under 1,000 euros
3 1,000 euros to under 1,500 euros
4 1,500 euros to under 2,000 euros
5 2,000 euros to under 2,500 euros
6 2,500 euros to under 3,000 euros
7 3,000 euros to under 3,500 euros
8 3,500 euros to under 4,000 euros
9 4,000 euros to under 4,500 euros
10 4,500 euros and more
11 not specified

Question S12
Take over federal state/region/location size from the sample.

Thank you very much for taking the time to participate in this interview!
